# A broad inhibitor of acyl-acyl carrier protein synthetases

**DOI:** 10.1016/j.bbrep.2023.101549

**Published:** 2023-09-23

**Authors:** Magdalena Todorinova, Joris Beld, Kara L. Jaremko

**Affiliations:** a Department of Chemistry, Hofstra University, Hempstead, NY, 11549, USA; b Department of Microbiology and Immunology, Drexel University College of Medicine, Philadelphia, PA, 19102, USA

**Keywords:** Acyl-acyl carrier protein synthetase, Fatty acid synthase, Fatty acid recycling, Inhibitor, Acyl carrier protein

## Abstract

The acyl-acyl carrier protein synthetase enzyme enables some bacteria to scavenge free fatty acids from the environment for direct use in lipids. This fatty acid recycling pathway can help pathogens circumvent fatty acid synthase (FAS) inhibition with established antibiotics and those in clinical development. AasS enzymes are surprisingly hard to identify as they show high sequence similarity to other adenylate forming enzymes, and only a handful have been correctly annotated to date. Four recently discovered AasS enzymes from Gram negative bacteria, *Chlamydia trachomatis, Neisseria gonorrhoeae,* and *Alistipes finegoldii*, form distinct clusters in protein sequence similarity networks and have varying substrate preferences. We previously synthesized C10-AMS, an inhibitor of AasS that mimics the acyl-AMP reaction intermediate. Here we tested its ability to be broadly applicable to enzymes in this class, and found it inhibits all four newly annotated AasS enzymes. C10-AMS therefore provides a tool to study the role of AasS in fatty acid recycling in pathogenic bacteria as well as offers a platform for antibiotic development.

## Highlights

• Four recently annotated acyl-acyl carrier protein synthetases were tested *in vitro*.
• These AasSs group in distinct clusters suggesting significant differences.
• All AasSs were active with EcACP and C_10_ substrates.
• C10-AMS inhibited all four AasS enzymes.

## Graphical abstract

**Figure:**
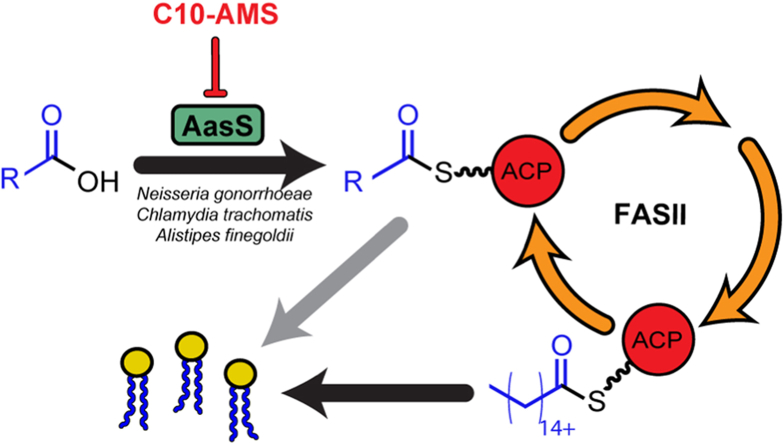

## 1 Introduction

**Fig. 1:**
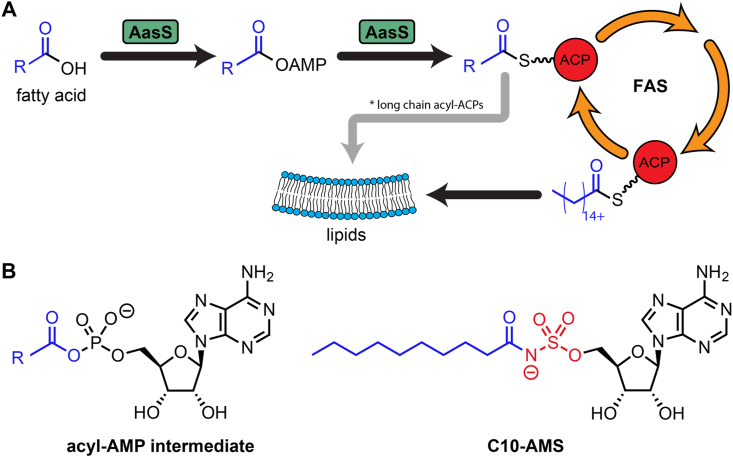
AasS activity and inhibitor design. (**A**) AasS can recycle fatty acids from the environment and load them onto the acyl carrier protein (ACP) in the fatty acid synthase (FAS) via a two-step reaction sequence. This allows long chain fatty acids to be incorporated into lipids, and short- and medium-chain fatty acids to be extended by FAS and then be incorporated into lipids. (**B**) The C10-AMS inhibitor structure mimics the acyl-AMP intermediate of the two-step reaction catalyzed by AasS. The inhibitor replaces the phosphate group with a non-hydrolyzable sulfamoyl linker (red) and mimics a C_10_ acyl substrate (blue). (For interpretation of the references to color in this figure legend, the reader is referred to the Web version of this article.)

Many bacterial pathogens salvage and utilize exogenous fatty acids, both as nutrient sources and direct building blocks [1]. Bacteria produce their own fatty acids via the fatty acid synthase (FAS) and these are essential to their existence, but the ability to recycle fatty acids can allow for energy to be spared for other cellular activities or help with survival during high-stress conditions [2]. One mechanism for environmental fatty acid recycling in bacteria is the loading of fatty acids onto the FAS acyl carrier protein (ACP) by acyl-acyl carrier protein synthetase (AasS) [1]. This enables them to be elongated or incorporated directly into lipids without first needing to be broken down (Fig. 1A). Since the FAS is a common antibacterial target for both established drugs and those in development [[3], [4], [5]], the potential of an organism to circumvent these drugs through AasS-enabled fatty acid recycling could prove problematic in targeting pathogenic organisms. For example, deletions in *Streptococcus agalactiae* FAS can be rescued by exogenous fatty acids, and we recently showed the rescue of *Vibrio cholerae* FAS inhibition using fatty acids [6,7]. The success of future antibiotics targeting FAS is therefore contingent upon understanding AasS activity and ultimately inhibiting it.

AasS is part of a large superfamily of adenylate-forming enzymes, a ubiquitous enzyme group including acyl-CoA ligases, non-ribosomal peptide synthase (NRPS) adenylation domains, tRNA synthetases, and NRPS-independent siderophore synthetases [8]. AasS enzymes function via a two-step mechanism. The first step is the ATP-dependent adenylation of free fatty acids to generate an acyl-AMP intermediate that binds tightly, but non-covalently, to the active site. The second step is the transesterification of the acyl moiety onto the thiol of the phosphopantetheinyl group of *holo-*ACP [1]. An AasS was first discovered in *Escherichia coli* by the Cronan lab [9]. However, this membrane-bound bifunctional enzyme was unstable *in vitro* and difficult to purify. A soluble AasS sub-type was later discovered in the bioluminescent bacterium *Vibrio harveyi,* and it has been rigorously studied since [[10], [11], [12]]. VhAasS can efficiently load chain lengths ranging from C_4_–C_18_ but is the most efficient with medium chain lengths centering around C_10_. Tight-binding inhibitors of adenylation domains and tRNA synthetases have been synthesized to mimic the AMP intermediate with a non-hydrolyzable linker [[13], [14], [15]]. We mimicked this approach and previously synthesized C10-AMS with three key regions: adenosine, C_10_ acyl chain, and a sulfamoyl linker, and showed it inhibits AasSs from *Vibrio harveyi* and *Vibrio cholerae* (Fig. 1B) [7].

Due to sequence and structure homology of AasS enzymes and acyl-CoA ligases, it is difficult to discover new AasS enzymes. As discovery strategies, others have noted that some bacteria contain fatty acids in their lipidome that they cannot biosynthesize, or that some bacteria can be grown in media containing unusual fatty acids that can then be found in their lipids after incubation [[16], [17], [18]]. By genome mining, researchers can subsequently identify putative AasSs or acyl-CoA ligases and characterize those *in vitro* or in bacteria via deletion. Recently, four new AasS enzymes were discovered in Gram negative bacteria [[16], [17], [18]]. *Chlamydia trachomatis* is a Gram-negative obligate intracellular bacterium that causes chlamydia, with an estimated 127 million new cases in 2016 worldwide [19]. AasC was discovered in the genome of *Chlamydia trachomatis* and verified *in vitro* with C_12_, C_14_, and C_16_ substrates [18]. *Neisseria gonorrhoeae* is a Gram-negative obligate human pathogen that causes gonorrhea, with >78 million new cases per year worldwide [20], and was found to encode AasN. Strains expressing AasN were able to activate and incorporate exogenously supplied C_12_–C_16_ substrates into phospholipids [16]. *Alistipes finegoldii* is a member of the Bacteroidetes, which are a major contributor to the gut microbiome. Perturbations in Bacteroidetes can be associated with obesity and diabetes [17]. *A. finegoldii* is able to use fatty acid nutrients in the gut environment to assemble membrane lipids via two AasS enzymes. AfAas1 and AfAas2 were identified by a bioinformatic search, cloning and expression, and subsequent *in vitro* testing with C_12_ and C_18:1_ substrates. AfAas1 selectively activated C_12_, whereas AfAas2 was most active with C_18:1_, but did activate C_12_ at one third the rate [17]. These AasSs do not share high sequence identity with those from Vibrionaceae [7]. They also show varying substrate loading capability and specificity ranging from C_12_ to C_18_ (Table 1), suggesting that perhaps specific chain length inhibitors are necessary to inhibit these enzymes. We therefore set out to test whether C10-AMS would be able to efficiently inhibit the catalytic activity of these different enzymes.

**Table 1:** Fatty acid substrates of AasS enzymes.

	Range of Fatty Acids Tested	Fatty Acids Successfully Loaded	Fatty Acid Preference
**VhAasS**	C_4_–C_18_	C_4_–C_18_	C_10_
**AasN**	C_12_, C_14_, C_16_	C_12_, C_14_, C_16_	n.d.
**AfAas1**	C_12_ and C_18:1_	C_12_	C_12_
**AfAas2**	C_12_ and C_18:1_	C_12_ and C_18:1_	C_18:1_
**AasC**	C_12_, C_14_, C_16_, C_18:1_	C_12_, C_14_, C_16_, C_18:1_	C_16_
**VcAasS**	C_10_	C_10_	n.d.

References for table data [7,10,12,[16], [17], [18]]; n.d. = not-determined.

## 2 Methods

### 2.1 Sequence analysis

Protein sequences for known AasSs were psi-blasted against the NCBI database and hits were combined in one fasta file. Sequence similarity networks (SSNs) were constructed using EFI-EST (parameters: filter value 100 and identity 75) [21] and visualized using Cytoscape [22].

### 2.2 Cloning

Expression constructs for AasN, AasC, AfAas1 and AfAas2 (ordered from Twist Biosciences) were constructed using isothermal assembly in pET16b and pET29a, as described [23].

### 2.3 Protein expression and purification

*Escherichia coli* ACP (EcACP), *Vibrio harveyi* AasS (VhAasS), *Neisseria gonorrhoeae* AasS (AasN), *Chlamydia trachomatis* AasS (AasC), *Alistipes finegoldii* AasSs (AfAas1 and AfAas2) and *Bacillus subtilus* Sfp were expressed as His_6_-tagged constructs in *E. coli* BL21[DE3]. 1 L cultures in Luria broth media (LB) with 100 mg/mL ampicillin or 50 mg/mL kanamycin were grown at 37 °C to an optical density at 600 nm (OD_600_) of 0.8, cooled to 16 °C, induced by the addition of isopropyl β-d-1-thiogalactopyranoside (IPTG) to 0.1 mM, and shaken for 18 h. Cells were harvested via centrifugation. The cell pellet was re-suspended in 30 mL buffer A (50 mM phosphate pH 7.5, 150 mM NaCl, 10% glycerol) with 1 mg/mL lysozyme, nutated at 4 °C for 1 h, and sonicated. The lysate was cleared via centrifugation at 15K x g for 30 min. The cleared lysate was batch-bound to 2 mL Ni resin and incubated for 1 h at 4 °C. The resin was washed with 100 mL buffer A followed by 100 mL buffer A containing 25 mM imidazole. The protein was eluted with buffer A containing 250 mM imidazole, desalted into buffer A, concentrated, and stored at −80 °C.

### 2.4 Synthesis of C10-AMS inhibitor

The C10-AMS inhibitor was synthesized according to previously published protocols [7].

### 2.5 Acyl carrier protein conversion and inhibition

A mixture of *apo*/*holo*-ACP (EcACP, 69 μM) was transformed into pure *holo*-ACP by incubation with Sfp (1.2 μM), MgCl_2_ (12.5 mM), TCEP (5 mM), and CoA (2 mM) in Buffer A for 18 h at 37 °C. Loading of fatty acids onto *holo*-ACP to produce acyl-ACP by AasS was performed with EcACP (30 μM), decanoic acid (1 mM), MgCl_2_ (12.5 mM), ATP (10 mM), TCEP (2.5 mM), and AasS (VhAasS – 2 μM; AasN – 25 μM; AfAas1 – 50 μM; AfAas2 – 50 μM; AasC – 50 μM) in Buffer A for 24 h at 37 °C. Inhibition was tested under the same conditions with the addition of C10-AMS (1 mM). We relied on conformationally sensitive urea-PAGE analysis to distinguish *apo*-, *holo*-, and acyl-ACP [24]. Samples (3 μg ACP each) were mixed with bromophenol blue load dye excluding SDS, loaded onto the gel, and run for 2 h at 180V. Protein bands were visualized with Bio-Safe Coomassie stain.

## 3 Results and discussion

The ACP substrate chosen for these studies was from *Escherichia coli* (EcACP)*,* a model organism to study bacterial pathways. VhAasS and VcAasS have been shown to be promiscuous for their ACP partners, efficiently loading decanoic acid (C_10_) onto EcACP [7,11]. When ACP is expressed as a His_6_-tagged construct in *E. coli* BL21[DE3], a mixture of *apo*- and *holo*-ACP is obtained. *Apo*-ACP is unmodified, while *holo*-ACP has been post-translationally modified with the 4′-phosphopantetheine moiety from CoA by a phosphopantetheinyl transferase. EcACP was converted completely into its *holo*-ACP form by a promiscuous phosphopantetheinyl transferase from *Bacillus subtilis,* preparing it to be a substrate for AasS [25].

**Fig. 2:**
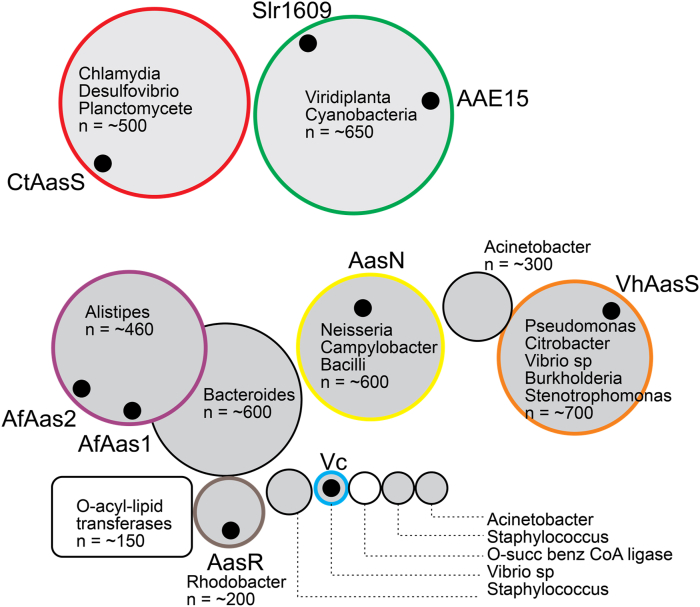
Protein sequence similarity network of AasS candidates. Sequences of characterized AasSs were used to assemble a list of >3000 protein sequences. Sequences were clustered using EFI-EST and visualized using Cytoscape. For simplicity, individual proteins are not shown but the combined number shown as “n” in each cluster. Most abundant genera in each cluster are shown.

In the absence of a structure of an AasS, a protein sequence similarity network was used to compare known AasSs (Fig. 2). AasSs group in distinct clusters, suggesting significant differences between these enzymes. The AasSs studied here include *Vibrio harveyi* AasS (VhAasS), *Neisseria gonorrhoeae* AasS (AasN), *Chlamydia trachomatis* AasS (AasC), and *Alistipes finegoldii* AasSs (AfAas1 and AfAas2) [10,[16], [17], [18]]. VhAasS was used as a control, and AasC and AasN represent examples from prolific and problematic pathogens. AfAas1 and AfAas2 have differing substrate preferences, and AfAas2 was most active with C_18:1_. They therefore represented a good model to test if C10-AMS was effective against AasS enzymes with varying and longer chain length preferences. The design of C10-AMS was modeled after known adenylation domain inhibitors, but decanoic acid is more apolar than adenylation domain substrates. Therefore, the final synthetic steps for C10-AMS were challenging due to solubility. Additionally, when screening AasS enzymes for loading of C_4_–C_18_ substrates, the solubility of C_14_–C_18_ in aqueous reaction conditions was significantly diminished. Therefore, inhibitor synthesis and application with longer chain lengths starts to become a real challenge. We hypothesized that while C_10_ may not be the natural substrate for these enzymes or the one they utilize most efficiently (Table 1), their binding pockets should have sufficient space to accommodate a C_10_ acyl chain. And although C_10_ may not bind as tightly to these enzymes, C10-AMS would since the acyl-AMP phosphodiester intermediate has a binding affinity 2-3 orders of magnitude times greater than the free fatty acid [26]. All enzymes were expressed as His_6_-tagged constructs in *E. coli* BL21[DE3] and purified by Ni-NTA affinity chromatography.

**Fig. 3:**
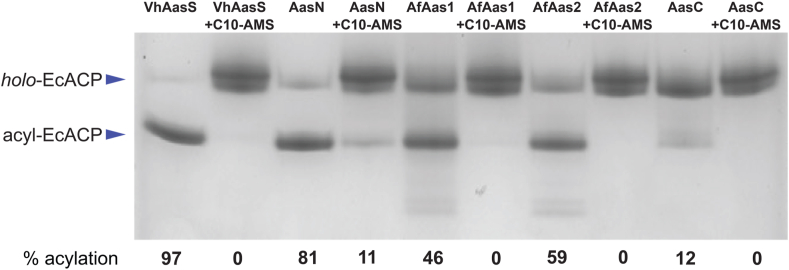
In vitro loading of holo-ACP with C_10_ catalyzed by AasSs and inhibition with C10-AMS. Urea-PAGE showing loading of C_10_ onto EcACP in odd lanes, and inhibition with C10-AMS in even lanes: VhAasS (lanes 1–2), AasN (lanes 3–4), AfAas1 (lanes 5–6), AfAas2 (lanes 7–8), AasC (lanes 9–10). 3 μg EcACP per sample. Percent acylation (%) for each reaction was determined by densitometry using ImageJ and is reported under each lane.

Thus far, AasC, AasN, AfAas1 and AfAas2 have only been tested with their cognate ACPs either *in vitro* or *in vivo*, and using substrates C_12_–C_18_ in chain length (Table 1). Here, EcACP and C_10_ were used as the substrates, and acyl loading was monitored by conformationally sensitive urea-PAGE gel. ACPs are small proteins, approximately 10 kDa, that have a characteristic four α-helical bundle and sequester their covalently-bound acyl chains. As ACP tightens around the attached acyl chain, it has an apparent smaller size and migrates further on urea-PAGE. Therefore, *holo*-ACP (not loaded with an acyl chain by AasS) and acyl-ACP (loaded with an acyl chain by AasS) can be distinguished by different gel shifts. All AasS enzymes tested were able to load C_10_ onto EcACP, with varying efficiencies determined by densitometry using ImageJ [27]: VhAasS (97%), AasN (81%), AfAas1 (46%), AfAas2 (59%), and AasC (12%) (Fig. 3). Some of the AasS enzymes were less active, even at much higher concentrations, but this can be attributed to using a non-cognate ACP substrate, using a C_10_ substrate that may not be preferred, or needing even higher enzyme concentrations to achieve 100% loading *in vitro*.

In parallel, the loading of C_10_ onto EcACP by each AasS was tested in the presence of the C10-AMS inhibitor. The inhibitor was added into the reaction mixture at the same time as C_10_ so that there was no pre-incubation with AasS. Inhibition of AasS activity was monitored by conformationally sensitive urea-PAGE, by measuring acyl-ACP (formed if inhibition was unsuccessful) by densitometry using ImageJ. Inhibition of acyl-ACP formation by C10-AMS was achieved 89% with AasN, and 100% with VhAasS, AfAas1, AfAas2, and AasC. Since we did not pre-incubate inhibitor with enzymes, it is possible that AasN was not completely inhibited due to a fast rate of loading substrate. Some trace extra bands in lanes 5 and lane 7 (Fig. 3) can be seen that are also acyl-ACP species, likely loaded with longer chain length fatty acids found in trace amounts in the reaction conditions by AfAas1 and AfAas2, highlighting varying chain length preferences. The formation of these species is additionally inhibited by C10-AMS as seen in lanes 6 and 8. C10-AMS acted as a competitive inhibitor for all five AasSs, showcasing the scope of C10-AMS to act upon AasSs from different clusters (Fig. 2) and with varying substrate preferences.

Only a few AasSs from bacteria have been characterized to date, but the presence of this enzyme is likely far more widespread. These synthetases show significant similarity with acyl-CoA ligases, which utilize the small molecule coenzyme A as their substrate rather than *holo*-ACP. Because these enzymes have a high homology, they cannot be distinguished by sequence alone. Adding to the confusion, many different names for each are used in the literature – acyl-CoA ligases are also called acyl-CoA synthetases, fatty acyl-CoA ligases (FACLs), and FabD homologs, while acyl-acyl carrier protein synthetases are also called fatty acyl-AMP ligases (FAALS), and even abbreviated AasS or Aas depending on the laboratory. We hypothesize that many AasS enzymes are misannotated, and many others have yet to be discovered. As more AasS enzymes are found, tools to study their native roles are needed. An unbiased cluster analysis of blastp hits in the entire nr database of the NCBI with known AasSs, provides excellent candidates in *Stenotrophomonas*, *Bacilli*, *Burkholderia*, *Campylobacter* and *Pseudomonas* species (Fig. 2). Several of these bacteria are emerging multidrug resistant pathogens. C10-AMS appears to be a wide-ranging inhibitor and can be used as a biochemical tool to study emerging enzymes in this class and their role in fatty acid recycling, as well as the relevance of fatty acid recycling at specific stages of infection.

Additionally, an ongoing debate is whether fatty acid biosynthesis/metabolism is a bona fide antibacterial target. For example, deletion in the FAS of *Streptococcus agalactiae* can be rescued by exogenous fatty acids, suggesting that the FAS is not a good target [6]. However, inhibition of the FAS by AFN-1252 in *Staphylococcus aureus* cannot be rescued by exogenous fatty acids [28]. A complicating factor in this work is that the fatty acid synthase not only produces acyl-ACPs for lipid biosynthesis, but is also involved in the biosynthesis of lipoic acid, biotin, quorum sensing molecules, and secondary metabolites. ACPs also have a regulatory role [29]. So to accurately mimic the complex acyl-ACP pool by providing exogenous fatty acids is not trivial in these studies. Still, in organisms like *Streptococcus agalactiae* and *Vibrio cholerae*, and more to come as AasSs are further annotated and studied, using both FAS inhibitors and fatty acid recycling enzyme inhibitors is an attractive strategy to fight bacteria. Here we show that C10-AMS is a potential lead compound for the development of new antibiotics to be used in conjunction with FAS inhibitors [30].

## Declarations and funding

The authors declare that the research was conducted in the absence of any commercial or financial relationships that could be construed as a potential conflict of interest.

JB thanks the Pennsylvania Department of Health for Commonwealth CURE funding.

## Author contributions

MT: Investigation, data curation; JB and KLJ: Conceptualization, validation, writing, visualization, supervision.

## Declaration of competing interest

The authors declare that they have no known competing financial interests or personal relationships that could have appeared to influence the work reported in this paper.
